# A Novel Electronic Record System for Documentation and Efficient Workflow for Community Health Workers: Development and Usability Study

**DOI:** 10.2196/52920

**Published:** 2024-04-01

**Authors:** Harshdeep Acharya, Kevin J Sykes, Ton Mirás Neira, Angela Scott, Christina M Pacheco, Matthew Sanner, Elizabeth Ablah, Kevin Oyowe, Edward F Ellerbeck, K Allen Greiner, Erin A Corriveau, Sarah Finocchario-Kessler

**Affiliations:** 1 Department of Internal Medicine Saint Peter's University Hospital New Brunswick, NJ United States; 2 Health and Wellness Center Baylor Scott and White Health Dallas, TX United States; 3 Department of Otolaryngology-Head and Neck Surgery University of Kansas Medical Center Kansas City, KS United States; 4 Department of Population Health University of Kansas Medical Center Kansas City, KS United States; 5 Department of Family Medicine & Community Health University of Kansas Medical Center Kansas City, KS United States; 6 Sanner Software Solutions Kansas City, KS United States; 7 Global Health Innovations Nairobi Kenya

**Keywords:** public health, database, community health worker, social determinants of health, health worker, health workers, CHW, CHWs, community-based, data collection, functionality, develop, development, EHR, EHRs, EMR, EMRs, dashboard, dashboards, health record, health records, documentation, medical record, medical records, equity, inequity, inequities

## Abstract

**Background:**

The COVID-19 pandemic added to the decades of evidence that public health institutions are routinely stretched beyond their capacity. Community health workers (CHWs) can be a crucial extension of public health resources to address health inequities, but systems to document CHW efforts are often fragmented and prone to unneeded redundancy, errors, and inefficiency.

**Objective:**

We sought to develop a more efficient data collection system for recording the wide range of community-based efforts performed by CHWs.

**Methods:**

The Communities Organizing to Promote Equity (COPE) project is an initiative to address health disparities across Kansas, in part, through the deployment of CHWs. Our team iteratively designed and refined the features of a novel data collection system for CHWs. Pilot tests with CHWs occurred over several months to ensure that the functionality supported their daily use. Following implementation of the database, procedures were set to sustain the collection of feedback from CHWs, community partners, and organizations with similar systems to continually modify the database to meet the needs of users. A continuous quality improvement process was conducted monthly to evaluate CHW performance; feedback was exchanged at team and individual levels regarding the continuous quality improvement results and opportunities for improvement. Further, a 15-item feedback survey was distributed to all 33 COPE CHWs and supervisors for assessing the feasibility of database features, accessibility, and overall satisfaction.

**Results:**

At launch, the database had 60 active users in 20 counties. Documented client interactions begin with needs assessments (modified versions of the Arizona Self-sufficiency Matrix and PRAPARE [Protocol for Responding to and Assessing Patient Assets, Risks, and Experiences]) and continue with the longitudinal tracking of progress toward goals. A user-specific automated alerts-based dashboard displays clients needing follow-up and upcoming events. The database contains over 55,000 documented encounters across more than 5079 clients. Available resources from over 2500 community organizations have been documented. Survey data indicated that 84% (27/32) of the respondents considered the overall navigation of the database as very easy. The majority of the respondents indicated they were overall very satisfied (14/32, 44%) or satisfied (15/32, 48%) with the database. Open-ended responses indicated the database features, documentation of community organizations and visual confirmation of consent form and data storage on a Health Insurance Portability and Accountability Act–compliant record system, improved client engagement, enrollment processes, and identification of resources.

**Conclusions:**

Our database extends beyond conventional electronic medical records and provides flexibility for ever-changing needs. The COPE database provides real-world data on CHW accomplishments, thereby improving the uniformity of data collection to enhance monitoring and evaluation. This database can serve as a model for community-based documentation systems and be adapted for use in other community settings.

## Introduction

The COVID-19 pandemic added to the decades of evidence that public health institutions are routinely stretched beyond their capacity, particularly in rural areas [1]. Local and state-level health department resource deficiencies reduce their ability to address health disparities [2]. Although the pandemic affected nearly everyone, communities of color, those residing in rural and frontier counties particularly around meatpacking plants, and other vulnerable populations disproportionately experienced COVID-19–related morbidity and mortality in addition to negative economic consequences [3-8]. Community health workers (CHWs) are a cost-effective intervention for achieving health equity [9]. They can establish trust with individuals and families adversely impacted by the social determinants of health (SDoH), sometimes called social risk factors, and effectively connect them to partner organizations that can address their needs.

The Communities Organizing to Promote Equity (COPE) project is an initiative to address health disparities in 20 counties across Kansas, in part, through the deployment of CHWs. This program also includes Local Health Equity Action Teams (LHEATs), which are community coalitions comprised of community residents, organizational leaders, and a cadre of CHWs who were hired for this project [10]. The goal of the COPE project is to mobilize communities to work together and prioritize local health equity issues, develop feasible strategies to address health equity barriers related to SDoH, collaborate with organizations in the community to strengthen access to services, and elevate the community’s voice in public health planning. One of the primary roles of COPE CHWs is developing partnerships with organizations in their respective counties. These partnerships enable CHWs to connect individuals and families with existing resources and services to address their needs. CHWs build community and individual capacity. They identify individuals who need assistance through community events, self-referrals, or referrals from organizations/clinics and actively work with these individuals to develop tailored plans to address their needs. CHWs are active members of their county’s LHEAT and provide insights from clients’ experiences to support the implementation of LHEAT-driven strategies and community-based events.

To document the project’s wide range of both community-based and client-based efforts, we reviewed 7 existing data tracking systems that accommodate CHW workflows, including electronic medical record (EMR) systems and available community-based documentation systems. Some of the limitations of the existing systems included (1) an exclusive focus on medical needs without documenting social risk factors like employment or housing status, (2) community-based platforms that focus on implementing a referral system but fail to track client goals and progress, (3) systems that lack a structure for progress evaluation and reporting, and (4) limitations on customization for specific project needs (eg, could not accommodate tracking of partnerships and events). In these existing systems, CHWs are often required to document in multiple platforms, resulting in fragmented data and increased redundancy, errors, and inefficiency.

Accurate documentation of CHWs’ influence on the community, public health, and health care processes is vital, particularly as states like Kansas consider Medicaid reimbursement models for CHWs [11]. We could not identify an existing platform, system, or database that efficiently captured specific information about partners at community organizations/facilities, events/activities, client demographics and outcomes, and longitudinal assessment of care plans [11]. Thus, we developed a data collection system capable of recording such information through a user-friendly interface with rigorous security measures for storing clients’ protected health information. We are unaware of other data tracking systems covering the full scope of CHW activities. This database includes details on partner organizations supporting medical and social needs, community outreach events, information about client encounters, and progress toward client’s goals. Further, we can customize workflows to account for unique geographic needs and resources. Finally, this system integrates all program activities into a single platform, simplifying program monitoring and evaluation.

In this paper, we describe the COPE database development process and outline the platform’s features and functionality. The lessons learned through this process and the values of this unique database can support further implementation and similar efforts to develop CHW client management systems.

## Methods

### Database Development

We applied the principles of human-centered design to develop a database covering the varied needs of the COPE project and serving as a data tracking, quality improvement, and evaluation platform [12]. The human-centered design approach allowed for iterative development informed by a multidisciplinary team of users. We engaged highly experienced CHWs with 20 years of experience (TMN and AS) and a physician well-versed in partnering with CHWs (HA) as the architects of the system’s functionality and user-friendly interface. The team conducted weekly meetings with the program engineers (MS and KO) to iteratively design and refine features. Pilot tests of the database features occurred over several months with 60 CHWs to ensure that the functionality supported their daily use and to identify areas for improvement. The engagement of COPE project management and evaluation staff ensured the pragmatic design of features to support quality improvement feedback to CHWs and facilitate the generation of automated monitoring reports for routine dissemination to community partners.

Three CHW experts mapped the requirements for data collection and created structured proformas indicating the different fields to be captured and their relevance to client outcomes and CHW productivity. For instance, a needs assessment was embedded within the database for CHWs to identify clients’ SDoH barriers, which CHWs would use to create goals for the client and allow them to track client progress toward goal completion, identify organizations providing services or commodities, and document reasons for goals not completed. These features enhanced the ability of CHWs to track clients and link them to critical community resources. Further, a tracking system was incorporated to monitor how CHWs were spending their time and to notify them when contact with a client was needed.

The plans were provided to 2 software developers who engineered the client and server-side applications. The proformas were modified as necessary to improve uniformity and ease of use. Consensus among the development team ensured the capture of valuable data while avoiding overdocumentation. These proformas were used to create mock-up screens envisioned for the database, and the engineering team mapped the pathway from a data entry interface to data transfer and storage. The system was designed to maintain client confidentiality and included user authentication requirements and a data partition to restrict access by county. Only CHWs who were user-assigned to a county had access to the client data within the county. Users must authenticate access through Microsoft’s Azure Active Directory to access the system. In addition to strong password requirements, multifactor authentication was required for each user account to add an extra layer of security. The application also included a time-out mechanism that logged the user out if they were determined to be inactive. The application was built using Microsoft’s Azure cloud hosting and used industry-standard practices for infrastructure—including but not limited to the encryption of data at rest and in-transit, network isolation, and the principle of least privilege for access management policies.

The COPE database team produced a minimum viable product (a beta version with essential components) and then integrated an iterative process incorporating feedback from CHWs into the development of the database at multiple stages (ie, a local development, staging, and production environment), which was sustained through the database implementation process to refine the product. Early development included a staging environment that was deployed to the cloud. The staging environment allowed the team to test the infrastructure and application code safely as new features were introduced to the database. Through this staging environment, CHWs interacting with the database provided feedback (eg, adapting need assessment items, altering information shown in client charts, adding an alert feature), which were integrated with the initial development of the database. Once essential functionality was established, a production environment was created for use by CHWs, supervisors, and administrators. Ongoing feedback via individual meetings, emails, or in-person communications was collected from CHWs and discussed during weekly meetings with the database engineer; once approved, changes were deployed to the production environment. To ensure Health Insurance Portability and Accountability Act (HIPAA) compliance, the production environment uses industry-standard network isolation, data encryption, and access control to protect against breaches and unauthorized use of the application or its data [13].

### Continuous Quality Improvement and Evaluation

To evaluate CHW performance, we employed a continuous quality improvement process [14]. This process allows for evaluating CHW workflows and accomplishments based on the number of clients enrolled, progress of client status and goals, and productivity measures. The continuous quality improvement plan is completed monthly for CHWs in each participating county. The supervisor provides feedback at the team and individual levels regarding the continuous quality improvement results and opportunities for improvement.

Primary partners in each county receive a quarterly report, which includes information on the number of partnerships entered, events completed, clients served, and individual client progress toward goals. Examples of local successes and challenges experienced by the LHEAT and CHW teams are also included. This frequent feedback allows each county to reflect on future goals and helps identify trends across the statewide project. Data exports can also be used to evaluate overall project outcomes. The COPE database was designed to be flexible, allowing the team to continue to innovate for future needs. Specifically, these established feedback systems with CHWs and partnering organizations and weekly meetings with the database engineer to implement technical changes supports the ability to adapt any feature, customize workflows, and alter alert systems, thereby maintaining flexibility of the database.

### Data Collection

A database feedback survey was conducted at 19 months after implementation (May 2022). A link to a 15-item REDCap (Research Electronic Data Capture) survey was emailed to all COPE CHWs and CHW supervisors. Close-ended questions consisted of demographic items (eg, age, education level, primary language) and assessed feasibility of database features and overall satisfaction. Respondents were asked to rank order the perceived importance of various database features. A Friedman test was conducted to determine whether participants had a differential rank-ordered preference for the database features. Open-ended questions assessed individuals’ perspectives toward accessibility and usability of the database and database features. Quantitative results were analyzed using SPSS statistics (version 29.0.0; IBM Corp). Qualitative results were analyzed using inductive content analysis [15].

### Ethics Approval

The protocol for this study was approved by the University of Kansas Medical Center institutional review board (STUDY00148455: COPE Project). This study was conducted in compliance with standards established by Good Clinical Practice, the International Council for Harmonisation, and the Declaration of Helsinki. Informed consent was obtained from all individual participants included in this study. Survey data were deidentified and stored on the University of Kansas Medical Center’s secure network drive specifically designated for storage of sensitive personal data. Data access will be restricted to those with appropriate institutional review board authorization and limited to principal and coinvestigators, statisticians, and data analysts involved in the analyses. All computer files and systems will be password-protected and accessible only by authorized personnel. The described data were collected as part of quality improvement in which CHW staff were asked to complete a feedback survey; no compensation was required.

## Results

### Database Development Process

The COPE database development process spanned 5 months and then went live for community pilot testing among COPE CHWs. The addition of auxiliary functions and refining of existing features continued for an additional 5 months. The development involved 613 cumulative working hours of the software developers and approximately 150 person-hours in active discussions with the COPE team. From the user perspective, CHWs each have access to enter client information, create assessments and goals, and update encounters and client status. They can also add and review events and partnerships within the database. CHW supervisors have additional rights to delete events, partnerships, client goals, or encounter information for flexibility purposes. This functionality facilitates quality improvement and provides a pathway for deduplication of records. At launch, the database had 60 active users in 20 counties, with the flexibility to expand.

### Database Components and Function

The COPE database is a secure, HIPAA-compliant, comprehensive, electronic, cloud-based application [16]. All user-specific logins are password secured with multifactor authentication and linked to county-specific access. Client documents such as consent forms and other protected health information are stored in a HIPAA-compliant environment [16]. CHWs and supervisors can locate client information, partners, and events pertinent to their daily work by using the web application interface. Data exports are packaged as CSV files to maintain compatibility with analysis software and are only available to system administrators. Exports can include or exclude identifiable data depending on analysis needs and the intended purpose for the data (eg, quality improvement reviews, evaluation analyses).

The database facilitates linkages to organizational partnerships by capturing contact information, facility location, services offered, and service areas. Once the partnership information is entered, users can build queries of organizations filtered by county, name, and services provided to facilitate client referrals. The database also includes data from community events such as the name and location of the hosting organization(s), event’s purpose, intended beneficiary population(s), and number of attendees. Event locations and partner addresses can be geocoded and overlayed with markers of community needs or vulnerability to guide the strategic deployment of resources to high-priority communities. During and after partner community events, the database is utilized for tracking client referrals and CHW engagement.

**Figure 1:**
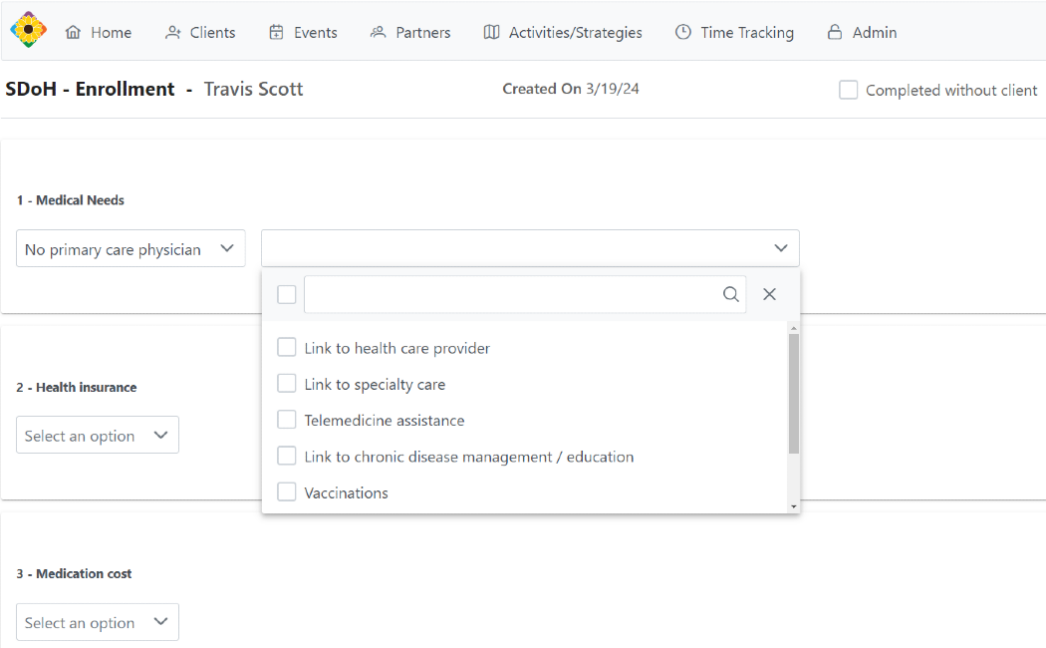
Screenshot of the staging environment used to train community health workers on entering and updating clients’ needs in the Communities Organizing to Promote Equity database. The data depicted were created for training purposes and are not from an actual client. SDoH: social determinants of health.

**Figure 2:**
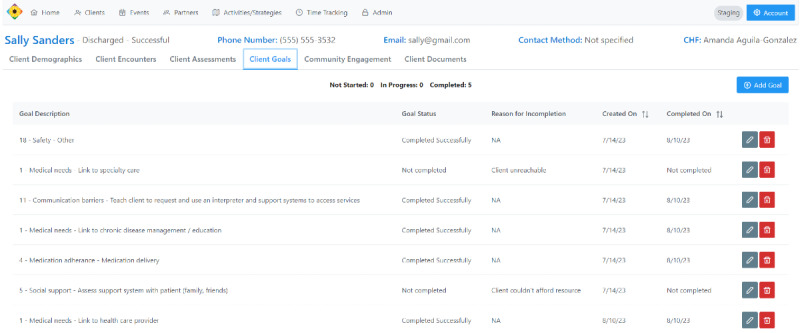
Screenshot of the staging environment used to train community health workers on how clients’ needs are populated as goals and tracked for follow-up and resolution in the Communities Organizing to Promote Equity database. The data depicted were created for training purposes and are not from an actual client. CHW: community health worker.

For client interactions, the system organizes client demographics, insurance status, and SDoH needs assessments (based on modified items from the Arizona Self-sufficiency Matrix and the PRAPARE [Protocol for Responding to and Assessing Patient Assets, Risks, and Experiences] tool; Figure 1) [17,18]. Once the client assessment is performed and entered in the database, client goals and care plans populate under the client’s chart, allowing CHWs to track progress and goal completion while working with clients (Figure 2). CHWs and clients establish time-bound goals and follow them to completion. Protocols were developed to support CHWs as they work with clients to prioritize needs. CHWs can document client referrals from partnering organizations and send referrals out to service organizations. This allows for monitoring the number of referrals, both into and out of organizations. Once applicable goals are completed, CHWs can close the loop with the referring organization or provider.

**Figure 3:**
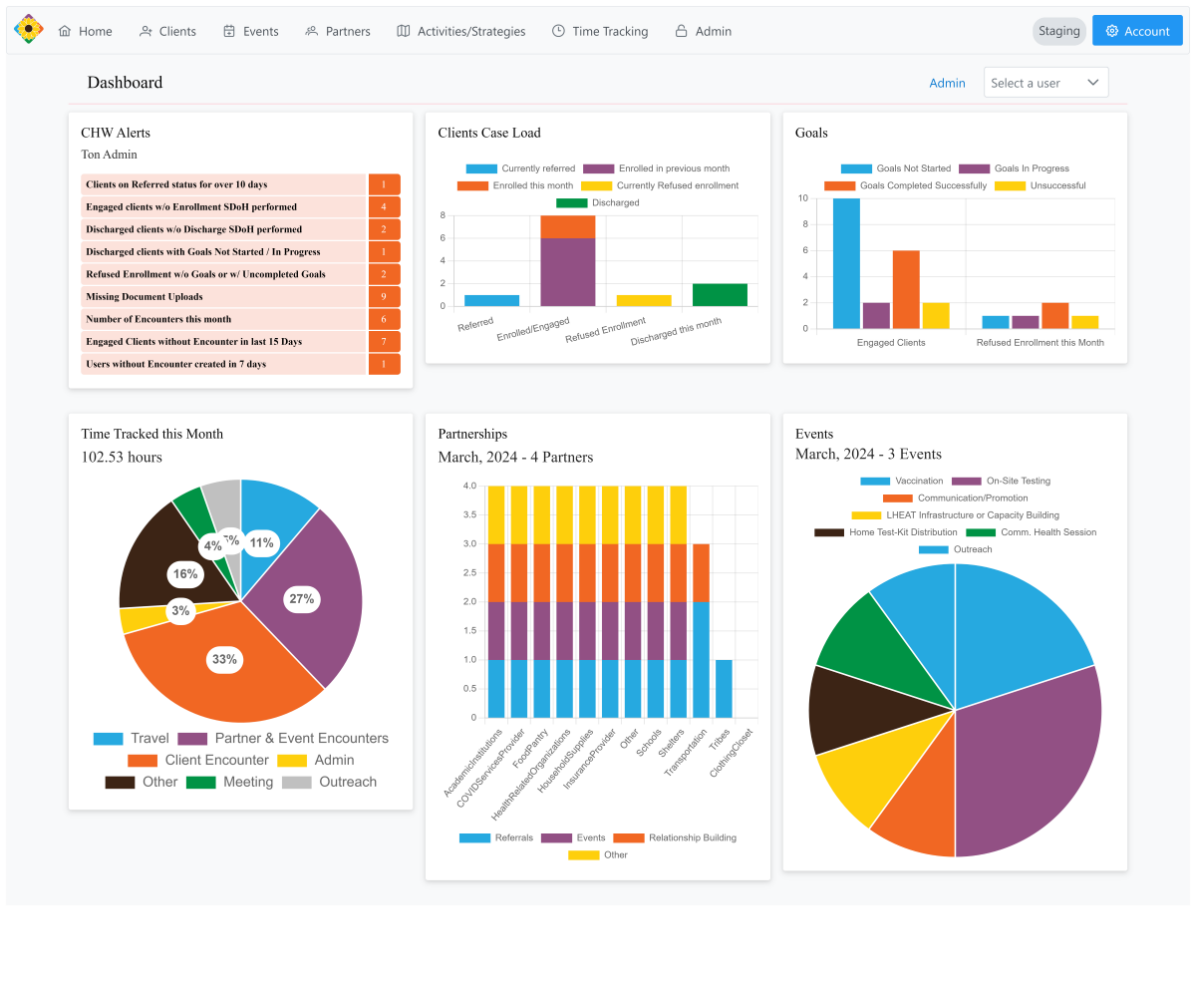
Screenshot of the staging environment used to train community health workers on the Communities Organizing to Promote Equity database dashboard summarizing community health worker alerts, client caseload, progress toward client goals, productivity time tracking, community partnerships established, and community-based outreach events completed. Data depicted were created to be representative of community health worker workloads for training purposes and are not from actual community health worker documentation. CHW: community health worker.

Algorithms and reports are generated to automate alerts for overdue actions and generate population-level summaries for real-time tracking and auditing. For efficient follow-up and priority setting, an automated alerts-based dashboard specific to the user’s account can notify the user of clients needing follow-up and upcoming events. The dashboard also builds a list of clients currently active, personalized to each CHW, including basic demographic and contact information, dates of the last contact, and the client’s status (eg, engaged, referred, discharged) (Figure 3).

### Database Usage and CHW Feedback

To date, over 60 CHWs have utilized the database across 20 counties in Kansas. The database contains over 55,000 documented encounters across more than 5079 clients. Available resources from over 2500 community organizations and partners have been documented. Nearly all current CHW and CHW supervisors (32/33, 97%) from the 20 COPE counties completed the survey. Of the respondents, 31% (10/32) were in the age range of 24-34 years, 25% (8/32) in the age range of 35-45 years, 25% (8/32) in the age range of 46-56 years, and 19% (6/32) in the age range of 57-67 years. CHW respondents reported a wide range of educational attainment with 9% (3/32) having a high school diploma or GED (general educational development) equivalent, 19% (6/32) some college, 22% (7/32) associate degree, 31% (10/32) bachelor’s degree, 16% (5/32) master’s degree, and 3% (1/32) having a doctorate’s degree. Most CHWs indicated English as their primary spoken language (24/32, 75%), with the remaining 25% (8/32) reporting Spanish as their primary language. CHWs reported spending an average of 7 hours each week on database documentation. Of the respondents, 84% (27/32) indicated that the overall navigation of the database was very easy. The majority of the respondents indicated that they were overall very satisfied (14/32, 44%) or satisfied (15/32, 48%) with the database.

Respondents ranked the following database features in order of most to least important: (1) dashboard (mean 3.26), (2) database alerts (mean 3.42), (3) assessments (mean 3.55), (4) goals (mean 3.97), (5) client demographics (mean 4.23), (6) time tracking (mean 5.68), (7) event charts (mean 5.90), and (8) partnership charts (mean 6.00). There was significant agreement on the rank-ordered preference for the database features (*χ*^2^_7_=49.5; *P*<.001) with a Kendall W of 0.228, which indicates moderate agreement between individuals on the preferable ordering of database features.

In their open-ended responses, CHWs described how the database helped build connections with community organizations by establishing them as partners. Documentation of community organization involvement and provision of these data to corresponding partners have translated into increased engagement at community events, increasing the resources available to community members. Feedback on event and partner data documentation has highlighted the need for improved workflows, specifically for client referrals during community events. Further, insights from CHWs led to the development of client transfer methods so that clients could be securely transferred within the database to another CHW, thereby enabling full access to client status and notes. This allowed clients to continue to progress toward goal completion. Lastly, a database component consistently mentioned by CHWs, further contributing to improved client enrollment processes, was the ability to provide clients with visible confirmation that all consent forms and data are stored in a HIPAA-compliant record system.

## Discussion

### Principal Results

The robust community-engagement strategies employed by the COPE project requires the development of a novel comprehensive database. The COPE database enhances the workflow and facilitates documentation for this innovative project by capturing process and evaluation data regarding individual and community needs and resources, thereby addressing health inequities and adverse SDoH. The data captured by this system extend beyond the conventional medical needs captured by existing EMRs while providing the required flexibility necessary for the ever-changing needs in communities. Finally, it provides a platform to document and quantify community-related information from the needs of individual members to the presence of key partner organizations positioned to meet these needs. The critical role CHWs play in improving community health outcomes and reducing the cost of health care is increasingly recognized [19-23]. However, little has been published on the process or outcomes of CHWs’ work. This project has contributed to the evidence base documenting the impact of CHWs on health inequities. Clients are empowered to complete goals with the assistance of CHWs. A client’s progress is thoroughly documented in the system, thereby establishing successful processes for connecting clients to services and identifying barriers to care. CHW documentation of client referrals (eg, from partnering organizations to service organizations) allows for monitoring of health barriers by demographics and locations and helps to close the loop with the referring partner. The database tracks client status (eg, referred, engaged, discharged), encounters with clients, progression toward goals, and client appointments with partnering organizations. The result is an efficient and effective case management support system to ensure clients receive the right care at the right time. This documentation demonstrates the impact and reach of CHWs, which is key to supporting health policy changes necessary for expanding and sustaining the CHW workforce through Medicaid or Medicare reimbursement models [24].

Data captured in the COPE database will enhance understanding of CHW workflow and CHWs’ ability to bridge gaps in current health systems. These data are critical to support the health policy changes necessary for expanding and sustaining the CHW workforce [25]. A multisectoral coalition with representation from state and local health departments, federally qualified health centers, community-based organizations, CHW leaders, and universities is advocating for sustainable models for the CHW workforce in Kansas through Medicaid and Medicare reimbursement [26]. Medicaid reimbursement for services provided by CHWs is a developing area of health policy and may require documentation systems like that of EMRs; however, these systems are not always applicable for the type of work conducted by CHWs, which may result in an undervaluation of their work and impact. As Kansas advances policy to support Medicaid reimbursement for CHWs, our database is being leveraged to track CHW performance and outcomes, as it is uniquely positioned to support CHW activities conducted outside the clinical setting [27]. Accordingly, this system could benefit community-based organizations or local health departments that employ CHWs.

### Limitations

There were limitations to concurrently developing a novel database platform while launching the COPE project. Given the timeline for system development, alternative strategies for tracking CHW activities were needed in the initial months of the project (eg, spreadsheets, forms stored on secure servers). These early data were transferred upon system deployment. Moreover, system customization and refinements were expedited by immediate and iterative feedback, starting with the first CHWs we hired. The database relies on the end user to close the loop with clients when provided with resources and to determine the effect of those resources. These outcome data are also provided to the referring partners. At this point, the database is not linked to an EMR system; however, the capacity to establish this linkage does exist. Interoperability with other systems implemented in federally qualified health centers and hospitals limited widespread implementation in the initial stages of launch. Most EMRs are not able to accommodate bidirectional data movement between the EMR and external databases. Consequently, depending on where a CHW is employed, this lack of EMR interoperability may create a double documentation issue for the end user.

Further, lack of infrastructure may cause potential limitations in the future. Currently, the project lacked support from a software firm, for example, EPIC or eClinicalWorks, which limited the applicability of the system’s interoperability. We anticipate interoperability with large-scale EMRs in the future, which would ideally allow the inclusion of needed documentation and information from CHWs into clinical care encounters, further facilitating the ability for dynamic navigation with CHWs and health care providers. With the expansion of the current systems’ novel features supporting medical and social needs, community outreach events, and progress toward client’s goals, clinic-based health care providers’ desire for closed-loop referral information can be improved to enhance community-centered care [28].

### Comparison With Prior Work

We acknowledge there are existing systems with rigorous features to capture essential aspects of CHW work; however, in the interest of avoiding multiple approaches to capturing the comprehensive range of CHW activities along with individual and project performance measures, we opted to build our own system. We could not identify an existing platform, system, or database that efficiently captured specific information about partners at community organizations/facilities, events/activities, client demographics and outcomes, and longitudinal assessment of care plans [11]. CHWs, the ultimate end users, designed and refined this system to optimize utility and performance. The current database system collaborates with organizations such as health departments, community-based organizations, health insurance providers, and federally qualified health centers in addition to consulting with similar platforms to incorporate feedback into the database design and discuss possibilities for interoperability of 2 or more systems.

### Sustainability

The COPE CHW project managers are continuously monitoring the operability of the database, including frequent review of user feedback and routine communication with system programmers to recommend adaptations and quickly resolve any system malfunctions. User feedback has been particularly helpful in the development and initial implementation phases in tailoring database components to meet CHW, supervisor, and administrator needs. Additionally, the database team collects feedback from partnering organizations and potential new clients looking to adopt the system for their organization. Processes are currently under development to streamline CHW feedback so that CHWs can directly send communications in real time to the database team regarding recommended changes or issues. These current methods, which have proven successful, will be sustained and enhanced to optimize database effectiveness by tailoring the product to organizations’ needs.

### Conclusions

The COPE database serves as a living directory of resources available to CHWs in Kansas. Comprehensive documentation of client referrals to and from partnering organizations allows for monitoring of health barriers experienced due to demographics, geographic location, and resources available. These data close the loop with the referring organization or providers and the resources they suggest to clients. Thus, the database provides real-world data on CHW successes and opportunities for improvement as they work toward health equity goals. Plans for expansion of the database in the future are currently under discussion, contracting with organizations to use the database, and maintaining collaborations to further adapt the system to improve client care coordination between providers and CHWs. Expansion of projects like this can help create valuable data sets for other states and counties to further support the broader implementation and documentation of CHW activities. The flexibility of the COPE database allows for tailoring the tool to fulfill the needs of similar projects and improve the uniformity of data collection to evaluate CHW outcomes. The COPE database can serve as a model for community-based documentation systems and can be adapted for use in other community settings.

## Abbreviations

CHW: community health worker
COPE: Communities Organizing to Promote Equity
EMR: electronic medical record
GED: general educational development
HIPAA: Health Insurance Portability and Accountability Act
LHEAT: Local Health Equity Action Team
PRAPARE: Protocol for Responding to and Assessing Patient Assets, Risks, and Experiences
REDCap: Research Electronic Data Capture
SDoH: social determinants of health

## Data Availability

There are no data to share publicly from this project. Data references in this manuscript are limited to quality assurance and improvement and would not be useful to those outside of the Communities Organizing to Promote Equity project.
